# Fast and Sensitive Determination of Bioflavonoids Using a New Analytical System Based on Label-Free Silver Triangular Nanoplates

**DOI:** 10.3390/s22030843

**Published:** 2022-01-22

**Authors:** Aleksei Furletov, Vladimir Apyari, Alexey Garshev, Stanislava Dmitrienko, Yury Zolotov

**Affiliations:** 1 Department of Chemistry, Lomonosov Moscow State University, Leninskie Gory, 1/3, 119991 Moscow, Russia; apyari@mail.ru (V.A.); alexey.garshev@gmail.com (A.G.); dmitrienko@analyt.chem.msu.ru (S.D.); zolotov@analyt.chem.msu.ru (Y.Z.); 2 Department of Materials Science, Lomonosov Moscow State University, Leninskie Gory, 1/73, 119991 Moscow, Russia; 3 Kurnakov Institute of General and Inorganic Chemistry, Russian Academy of Sciences, Leninsky Avenue, 31, 119991 Moscow, Russia

**Keywords:** antioxidants, bioflavonoids, nanoparticles, optical sensors, reducing agents, silver triangular nanoplates, spectrophotometry, surface plasmon resonance

## Abstract

Optical sensors based on silver triangular nanoplates (AgTNPs) are insufficiently studied as probes for the spectrophotometric determination of biologically active compounds. In the present article, an interaction between label-free AgTNPs and bioflavonoids in the presence of silver(I) ions was assessed to outline the possibilities of AgTNPs as a colorimetric probe for the fast and sensitive determination of bioflavonoids. It is shown that the interaction was accompanied by a bathochromic shift of the local surface plasmon resonance band of nanoparticles and an increase in its intensity. Seven bioflavonoids differing in their structure were tested. The influence of the structure of analytes and the main external factors on the analytical signal is discussed in detail. It was found that the detection limits of bioflavonoids in the selected optimal conditions increased in the series morin < rutin = quercetin < taxifolin and came to 0.9, 1.2, 1.2, and 2.0 μmol L^−1^, respectively. Chrysin, naringenin, and naringin were found not to affect the spectral characteristics of AgTNPs. The suggested approach was applied for the spectrophotometric determination of flavonoids in pharmaceuticals and onion peel.

## 1. Introduction

Bioflavonoids are major secondary metabolism compounds that occur naturally in various fruits and medical plants. Consequently, they can be considered as an important factor to be estimated in agriculture and food manufacturing, since their content strongly depends on the quality of fruits and plants [1]. Furthermore, bioflavonoids exhibit a vast spectrum of biological activity and have a direct impact on the functioning of living organisms [2,3,4,5,6].

A large number of analytical methods have been proposed for bioflavonoid detection in different samples. These methods include molecular spectroscopy [7,8,9,10,11], high-performance liquid chromatography [12,13,14,15,16,17], capillary electrophoresis [18,19], and different electrochemical methods, mainly voltammetry [20,21,22]. Generally, molecular spectroscopy and electrochemical methods can be used for fast, simple, and nonselective assessment of the content of flavonoids in samples, while chromatographic approaches allow the determination of individual bioflavonoids with a sensitivity that is several orders of magnitude higher than the sensitivity of molecular spectroscopy. Despite sometimes good analytical characteristics, the vast majority of these methods are expensive, complex in operation, or reagent-consuming. Moreover, a sample preparation step is often required to prepare the sample into a form ready for the analysis. Usually, for this purpose, samples are subjected to various options for liquid–liquid extraction (MAE, UAE, PLE, SPE, SFE), which is not environmentally friendly due to the need to use large volumes of toxic solvents; this also significantly complicates the analysis procedure [1]. Thus, the search for inexpensive, simple, environmentally friendly, sensitive, and selective methods for the determination of bioflavonoids is an essential task of analytical chemistry.

A good opportunity seems to be provided by nanoparticle-based approaches. Noble-metal nanoparticles, including label-free silver triangular nanoparticles (AgTNPs), are proposed for compound quantitation using spectral methods of analysis [23,24,25,26,27,28]. The most common principle underlying nanoparticle-based processes is their colloidal aggregation occurring for many types irrespective of their nature [29]. For metal nanoparticles, this usually results in drastic changes in their optical properties that can be easily detected using UV/Vis spectrophotometry, digital colorimetry, or even the naked eye. Some papers reported on the usage of silver nanoparticles for the determination of reducing agents [30,31]. However, to the best of our knowledge, the published papers did not consider silver triangular nanoplates as colorimetric reagents, although their potential in optical spectroscopy methods is clearly not fully disclosed.

In the present study, characterization of label-free AgTNPs in the interaction with seven bioflavonoids differing in their structure in the presence of silver(I) nitrate was assessed to outline the possibilities of AgTNPs as a colorimetric probe for the determination of these biologically active compounds.

## 2. Materials and Methods

### 2.1. Reagents and Instruments

In the present study, the following reagents were used: acetic acid (IREA 2000, Moscow, Russia, pure grade), hydrogen peroxide (Sigma-Aldrich, St. Louis, MO, USA, 30 wt.% in H_2_O, ACS), 1-ethenylpyrrolidin-2-one (Acros Organics, Waltham, MA, USA, M.W. 58,000 g mol^−1^, 99%), chrysin (Acros Organics, Waltham, MA, USA, 99+%), morin hydrate (Acros Organics, Waltham, MA, USA, pure grade), naringenin (Acros Organics, Waltham, MA, USA, 97%), naringin (Acros Organics, Waltham, MA, USA, 97%), quercetin dihydrate (Sigma-Aldrich, St. Louis, MO, USA, ≥98% (HPLC)), rutin (Acros Organics, Waltham, MA, USA, 97+%), silver nitrate (PZTsM-Vtormet, Moscow, Russia, analytical grade), sodium borohydride (Acros Organics, Waltham, MA, USA, 99%), sodium citrate (Sigma-Aldrich, St. Louis, MO, USA, ≥99.5%), sodium hydroxide (Reachim, Moscow, Russia, pure grade), sodium metabisulfite (Acros Organics, Waltham, MA, USA, pure grade), and taxifolin (Flamena, Moscow, Russia, 92%). Stock solutions of bioflavonoids were prepared by dissolving their weighed portions in 0.1 mol L^−1^ NaOH. Stock solutions of other substances were prepared by dissolving their weighed portions or aliquots in deionized water obtained with a Millipore Simplicity water purification system (Merck Millipore, Burlington, MA, USA). Working solutions of all substances were prepared by diluting the stock solution immediately before use.

Solids were weighed using an Adventurer analytical balance (OHAUS, Parsippany, NJ, USA). Discovery Comfort automatic single-channel pipet-dispensers of variable volume (HTL, Warsaw, Poland) were used to take aliquots of liquids. Absorption spectra of nanoparticles were recorded in standard quartz cuvettes with the optical path length of 1 cm using a SF-104 spectrophotometer (Akvilon, Moscow, Russia). The pH values of working solutions were measured using an Ekspert 001 pH-meter (Ekoniks Ekspert, Moscow, Russia).

Transmission electron microscopy (TEM) images of AgTNPs were recorded with a Libra 200 microscope (Carl Zeiss, Jena, Germany) with a thermal field-emission cathode at the accelerating voltage of 200 kV. Samples of studied nanoparticles were deposited onto a copper grid support with a formvar film covered by amorphous carbon Formvar^®^/Carbon Reinforced Copper Grids 3440C-MB (SPI, West Chester, PA, USA).

Chromatographic determination of bioflavonoids was performed using a Tsvet Yauza system (Khimavtomatika, Voronezh, Russia) equipped with an amperometric detector (E = 1.2 V). A chromatographic column Gemini 5U C18 (30 × 2 mm) was used. The mobile phase contained acetonitrile (HPLC) and 0.1% aqueous phosphoric acid solution in a ratio of 1:3. The flow rate of the mobile phase was equal to 0.40 mL min^−1^. The sample volume was equal to 0.8 mL, and injection of the samples was performed with a 20 μL loop.

### 2.2. AgTNPs Synthesis

Synthesis of AgTNPs was performed as described in [32] with minor modifications. The glassware used was cleaned with freshly prepared aqua regia, thoroughly rinsed with distilled water, and dried. Then, 4.8 mL of 1.04 mmol L^−1^ AgNO_3_ aqueous solution, 2.3 mL of 1% sodium citrate aqueous solution, 0.6 mL of 20 g L^−1^ 1-ethenylpyrrolidin-2-one aqueous solution, and 1.2 mL of 3% H_2_O_2_ aqueous solution were successively added into the reaction mixture under vigorous stirring. A 1.0 mL aliquot of a freshly prepared 35 mmol L^−1^ NaBH_4_ aqueous solution was added dropwise into the mixture under stirring. The mixture became a pale-yellow color, which half an hour later abruptly changed into intense emerald green and then became blue violet. At this point, the stirring was stopped. The AgTNP colloidal solution was stored at room temperature. The calculated final concentration of AgTNPs in the solution was 56 mg L^−1^ (0.52 mmol L^−1^ in terms of Ag atoms).

### 2.3. Procedure

To study the interaction between AgTNPs and bioflavonoids in the presence of silver(I) nitrate, 0.78 mL of AgTNP aqueous solution, various amounts (0.2–1.2 µmol) of bioflavonoids, and 0.80 mL of 1 mmol L^−1^ sodium metabisulfite solution were added into polypropylene test-tubes. Then, 0.20 mL of 10 mmol L^−1^ silver(I) nitrate aqueous solution was added into each test-tube. Last of all, acetate buffer solution (pH 6.5) was added to the reaction system to the final volume of 10.0 mL. After 20 min, the absorption spectra of AgTNPs were recorded.

### 2.4. Sample Pretreatment

Ascorutin pharmaceutical drug (Pharmstandard JSC, Dolgoprudny, Russia), ethanol tinctures of hawthorn (Flora Kavkaza JSC, Pregradnaya, Russia) and calendula (MosPharma JSC, Moscow, Russia), and onion were purchased from local suppliers. Both the hawthorn and the calendula tinctures were used without any special preparation.

A stock solution of the Ascorutin pharmaceutical drug was prepared according to the following procedure: 30 mL of distilled water was added to the one crushed tablet, after which the mixture was shaken on a mechanical vibrating mixer for 15 min. At the end of shaking, the precipitate containing rutin, almost insoluble in water, was separated from the solution by centrifugation (4000 rpm, 5 min) and dissolved in 0.1 mol L^−1^ NaOH.

The onion peel extracts were prepared according to [10] with slight modifications. The peel was detached from the onion and crushed in small pieces. Then, 0.5 g of the sample was extracted with 40 mL of 50% ethanol in an ultrasonic bath at 60 °C for 30 min. The extract was filtered and diluted several times with distilled water to get a desired concentration of a final solution.

## 3. Results

### 3.1. Interaction between Label-Free Silver Triangular Nanoplates and Bioflavonoids in the Presence of Silver(I) Nitrate

**Scheme 1:**
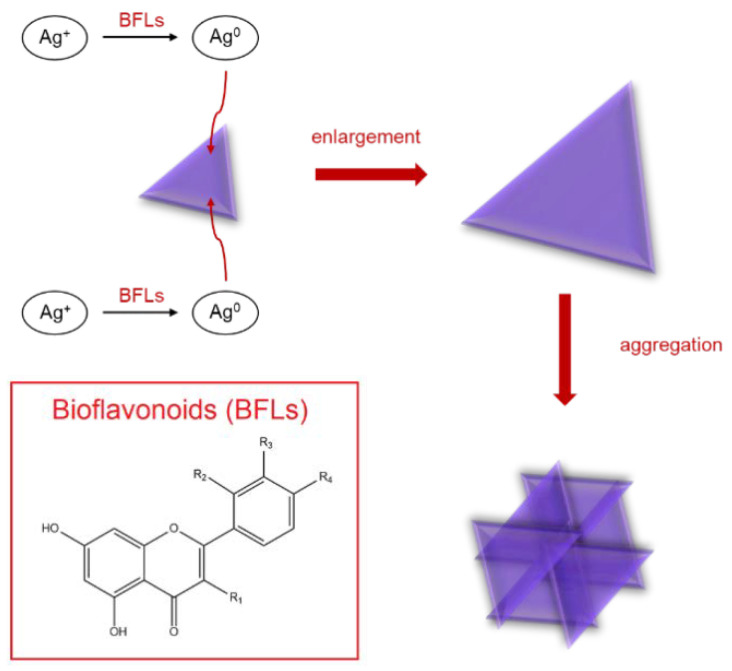
Principle of the proposed approach for the determination of bioflavonoids (description is given in the text).

**Figure 1:**
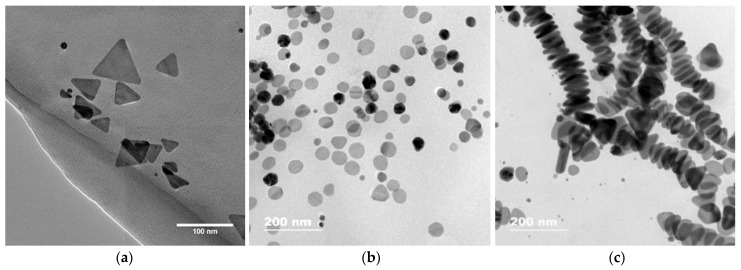
TEM images of studied nanoparticles before (**a**) and after (**b**,**c**) the interaction with quercetin in the presence of silver(I) nitrate: pH = 6.5 (**a**,**c**), 10.8 (**b**); *c*(AgTNPs) = 0.04 mmol Ag L^−1^, *c*(quercetin) = 60 μmol L^−1^, *c*(Na_2_S_2_O_5_) = 80 μmol L^−1^, *c*(AgNO_3_) = 0.20 mmol L^−1^, *t* = 20 min.

The interaction between AgTNPs and bioflavonoids in the presence of silver(I) ions does not seem to be a simple process. We assume that the interaction mechanism consists of two successive stages (Scheme 1). The first one includes reduction of silver(I) ions to metallic silver under the action of bioflavonoids and its deposition on the surface of the seed AgTNPs [33,34], which affect their size and morphology (Figure 1). The second stage includes the formation of irregular and plane-to-plane AgTNPs aggregates, whose existence was also confirmed by considering the TEM images of AgTNPs after the interaction (Figure 1c). Most likely, aggregates are formed due to a decrease in the thickness of the electric double layer, which leads to a decrease in the electrokinetic potential of nanoparticles and a loss of aggregative stability of the system.

**Figure 2:**
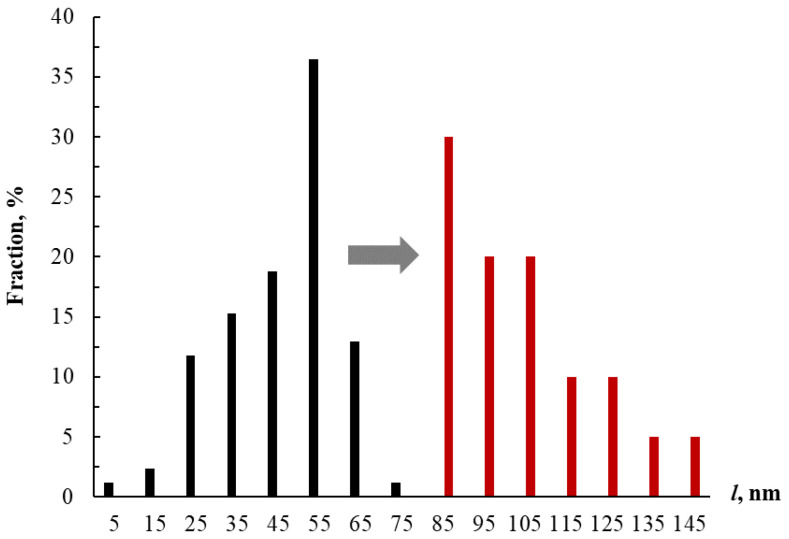
Histogram of silver triangular nanoplates distribution along the average edge length before (columns in black) and after the interaction (columns in red) with quercetin in the presence of silver(I) nitrate. *c*(AgTNPs) = 0.04 mmol Ag L^–1^, *c*(quercetin) = 60 μmol L^–1^, *c*(Na_2_S_2_O_5_) = 80 μmol L^–1^, *c*(AgNO_3_) = 0.20 mmol L^–1^, pH 6.5, *t* = 20 min.

A histogram of AgTNP distribution along the average edge length before and after the interaction with quercetin in the presence of silver(I) nitrate was plotted (Figure 2). This histogram was calculated from over 100 nanoparticle TEM images. It is easy to see that the average edge length of silver triangular nanoplates increased nearly twofold after the interaction, which convincingly confirms our assumption that silver is deposited on the AgTNP surface.

**Figure 3:**
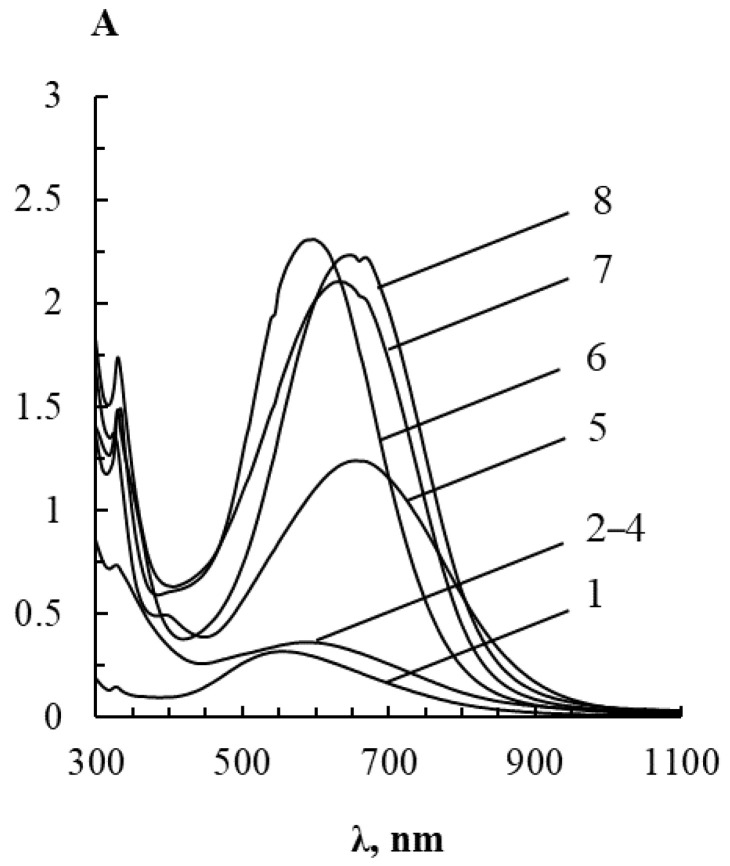
Absorption spectra of silver triangular nanoplates before (1) and after the interaction with chrysin (2), naringin (3), naringenin (4), taxifolin (5), morin (6), quercetin (7) and rutin (8) in the presence of silver(I) nitrate. *c*(bioflavonoids) = 100 μmol L^–1^; *c*(AgTNPs) = 0.04 mmol Ag L^–1^, *c*(Na_2_S_2_O_5_) = 80 μmol L^–1^, *c*(AgNO_3_) = 0.20 mmol L^–1^, pH 6.5, *t* = 20 min.

It is well known that optical properties of silver triangular nanoplates strongly depends on their size and aggregation state [35]. In this sense, reduction activity and the structure of bioflavonoids may drastically affect their ability to interact with AgTNPs. In the present study, seven bioflavonoids differing in their structure and reduction activity were tested (Table 1). Absorption spectra of AgTNPs before and after interaction with bioflavonoids in the presence of AgNO_3_ are shown in Figure 3. One can see that chrysin, naringenin and naringin do not to affect the spectral characteristics of AgTNPs. The probable reason for this fact is that these three bioflavonoids have relatively high redox potentials and cannot act as reducing agents in the reaction with silver(I) nitrate.

**Figure 4:**
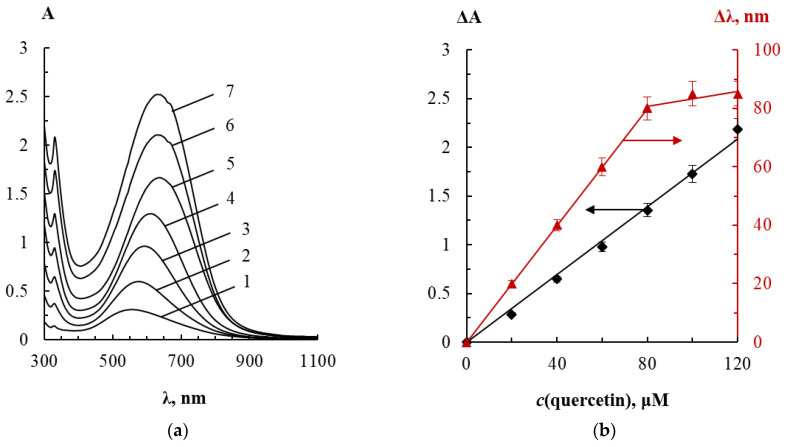
(**a**) Absorption spectra of silver triangular nanoplates before (1) and after (2–7) the interaction with quercetin in the presence of silver(I) nitrate at different analyte concentrations. *c*(quercetin), μmol L^−1^ = 0 (1), 20 (2), 40 (3), 60 (4), 80 (5), 100 (6), 120 (7); *c*(AgTNPs) = 0.04 mmol Ag L^−1^, *c*(Na_2_S_2_O_5_) = 80 μmol L^−1^, *c*(AgNO_3_) = 0.20 mmol L^−1^, pH 6.5, *t* = 20 min. (**b**) Dependences ΔA = *f*(*c*(quercetin), μmol L^−1^) and Δλ, nm = *f*(*c*(quercetin), μmol L^−1^). *c*(AgTNPs) = 0.04 mmol Ag L^−1^, *c*(Na_2_S_2_O_5_) = 80 μmol L^−1^, *c*(AgNO_3_) = 0.20 mmol L^−1^, pH 6.5, *t* = 20 min.

Meanwhile, it was found that the interaction between AgTNPs and morin, rutin, quercetin, and taxifolin in the presence of silver(I) nitrate is accompanied by both a bathochromic shift of the AgTNP LSPR band and a significant increase in its intensity (Figure 4a). On the basis of these facts, one can conclude that the interaction process is facilitated by low values of redox potentials and the presence of two hydroxyl groups located in the B ring of a flavonoid molecule.

To select the analytical signal, the dependences ΔA = *f*(*c*(bioflavonoid), μmol L^−1^) and Δλ, nm = *f*(*c*(bioflavonoid), μmol L^−1^) were considered, where ΔA is the increase in absorbance at the LSPR band maximum, and Δλ (nm) is the wavelength shift measured between the maxima of the corresponding spectra. These dependencies for quercetin are presented in Figure 4b. The detection limit of quercetin calculated from the first dependence turned out to be slightly lower due to the better reproducibility of the analytical signal of the blank experiment. Moreover, the linearity range for it was also wider. Therefore, it was proposed to use ΔA as an analytical signal for the spectrophotometric determination of bioflavonoids.

To improve the analytical performance of the proposed approach, the influence of AgTNP concentration, pH, interaction time, and silver(I) nitrate concentration on the analytical signal was studied in detail using the example of quercetin.

#### 3.1.1. Effect of Nanoparticle Concentration

**Figure 5:**
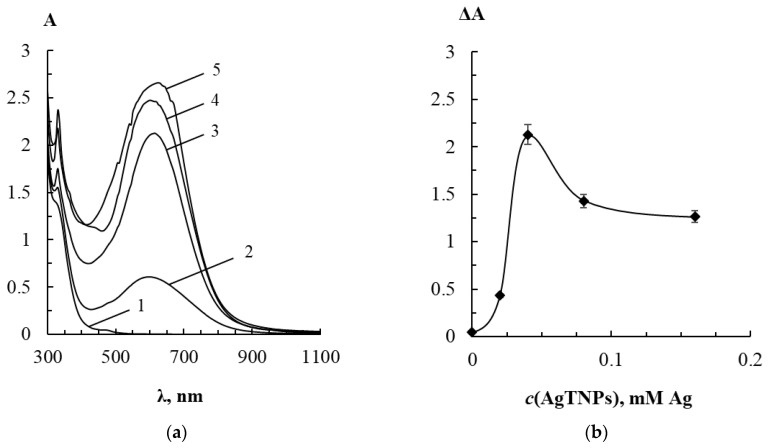
(**a**) Absorption spectra of silver triangular nanoplates after the interaction with quercetin in the presence of silver(I) nitrate at different AgTNP concentrations. *c*(AgTNPs), mmol Ag L^−1^ = 0 (1), 0.02 (2), 0.04 (3), 0.08 (4), 0.16 (5); *c*(quercetin) = 120 μmol L^−1^, *c*(Na_2_S_2_O_5_) = 80 μmol L^−1^, *c*(AgNO_3_) = 0.20 mmol L^−1^, pH 6.5, *t* = 20 min. (**b**) Dependence of the analytical signal (ΔA) on AgTNP concentration. *c*(quercetin) = 120 μmol L^−1^, *c*(Na_2_S_2_O_5_) = 80 μmol L^−1^, *c*(AgNO_3_) = 0.20 mmol L^−1^, pH 6.5, *t* = 20 min.

The effect of AgTNP concentration was evaluated within the range of 0–0.16 mmol Ag L^−1^. Absorption spectra of AgTNPs after their interaction with quercetin in the presence of silver(I) nitrate, as well as the dependence of ΔA on AgTNPs concentration, are presented in Figure 5. One can see that, in the case of the 0.16 mmol Ag L^−1^ AgTNP solution, the LSPR band of nanoparticles had a fuzzy maximum due to the presence of information noise, probably related to UV/Vis spectrometer limitations. This circumstance made it difficult to correctly detect the analytical signal. The spectra corresponding to 0.04 mmol Ag L^−1^ and 0.08 mmol Ag L^−1^ had a clear LSPR band maximum and were quite similar in shape and intensity. It was found that both the largest bathochromic shift of the LSPR band and the largest AgTNP absorbance change were achieved in the case of the 0.04 mmol Ag L^−1^ AgTNP solution. Thus, the AgTNP concentration of 0.04 mmol Ag L^−1^ was chosen for further experiments.

#### 3.1.2. Effect of pH

**Figure 6:**
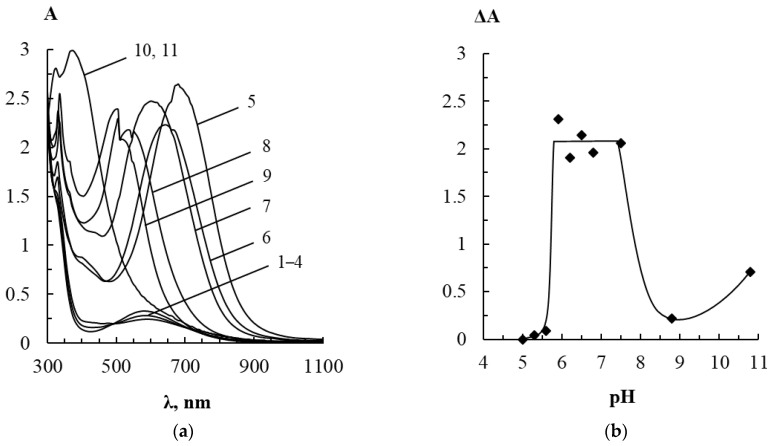
(**a**) Absorption spectra of silver triangular nanoplates before (1) and after (2–11) the interaction with quercetin in the presence of silver(I) nitrate at different pH. pH 6.5 (1); 5.0 (2), 5.3 (3), 5.6 (4), 5.9 (5), 6.2 (6), 6.5 (7), 6.8 (8), 7.5 (9), 8.8 (10), 10.8 (11); *c*(AgTNPs) = 0.04 mmol Ag L^−1^, *c*(quercetin) = 120 μmol L^−1^, *c*(Na_2_S_2_O_5_) = 80 μmol L^−1^, *c*(AgNO_3_) = 0.20 mmol L^−1^, *t* = 20 min. (**b**) Dependence of the analytical signal (ΔA) on pH. *c*(AgTNPs) = 0.04 mmol Ag L^−1^, *c*(quercetin) = 120 μmol L^−1^, *c*(Na_2_S_2_O_5_) = 80 μmol L^−1^, *c*(AgNO_3_) = 0.20 mmol L^−1^, *t* = 20 min.

pH plays a key role in modifying charge state of AgTNPs, thus affecting their aggregative stability, as well as the reductive ability of flavonoids. Absorption spectra of AgTNPs after their interaction with quercetin in the presence of silver(I) nitrate at different pH, as well as the dependence of the change in LSPR band intensity on pH, are presented in Figure 6. It was found that the biggest change in the AgTNP LSPR band intensity was observed in the pH range of 6.0–7.5. If one of the boundary values is chosen for the experiments, the slightest change in pH can lead to a strong decrease in the analytical signal. In this regard, pH 6.5 was chosen as a value approximately corresponding to the middle of the above range.

It should be noted that no bathochromic shift of the AgTNP LSPR band was observed in the alkaline medium. On the contrary, there were both a hypsochromic shift of this band and a lower increase in its intensity. It should also be emphasized that, in addition to the LSPR band in the wavelength range of 400–410 nm, a local maximum of the LSPR band in the wavelength range of 600–620 nm appeared in the absorption spectra of nanoparticles, indicating that AgTNPs remained unchanged or underwent slight changes in the process under consideration. These results seem to indicate that, in the alkaline medium, AgTNPs were not coated with a layer of silver, but rather spherical and other near round-shaped silver nanoparticles were formed in solution (Figure 1b). This process was also facilitated by a significant decrease in the redox potential of bioflavonoids at high pH, which increased their reductive ability.

#### 3.1.3. Effect of Interaction Time

**Figure 7:**
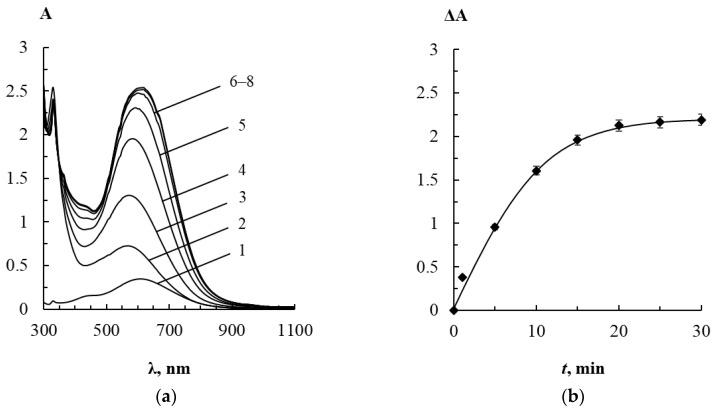
(**a**) Absorption spectra of silver triangular nanoplates before (1) and after (2–8) the interaction with quercetin in the presence of silver(I) nitrate at different interaction time. *t*, min = 0 (1), 1 (2), 5 (3), 10 (4), 15 (5), 20 (6), 25 (7), 30 (8); *c*(AgTNPs) = 0.04 mmol Ag L^−1^, *c*(quercetin) = 120 μmol L^−1^, *c*(Na_2_S_2_O_5_) = 80 μmol L^−1^, *c*(AgNO_3_) = 0.20 mmol L^−1^, pH 6.5. (**b**) Dependence of the analytical signal (ΔA) on the interaction time. *c*(AgTNPs) = 0.04 mmol Ag L^−1^, *c*(quercetin) = 120 μmol L^−1^, *c*(Na_2_S_2_O_5_) = 80 μmol L^−1^, *c*(AgNO_3_) = 0.20 mmol L^−1^, pH 6.5.

To study the effect of interaction time, the absorption spectra of AgTNPs were recorded 0, 1, 5, 10, 15, 20, 25, and 30 min after mixing of the reagents (Figure 7a). A gradual shift of the AgTNP LSPR band toward longer wavelengths and an increase in its intensity over time were recorded during the first 20 min.

The dependence of the analytical signal on the interaction time is presented in Figure 7b. It can be seen that, for a solution containing 120 μmol L^−1^ quercetin, 20 min is enough to complete the reduction of silver(I) ions. Therefore, this time was chosen to hold the solutions before recording the absorption spectra of nanoparticles.

#### 3.1.4. Effect of Silver(I) Nitrate Concentration

**Figure 8:**
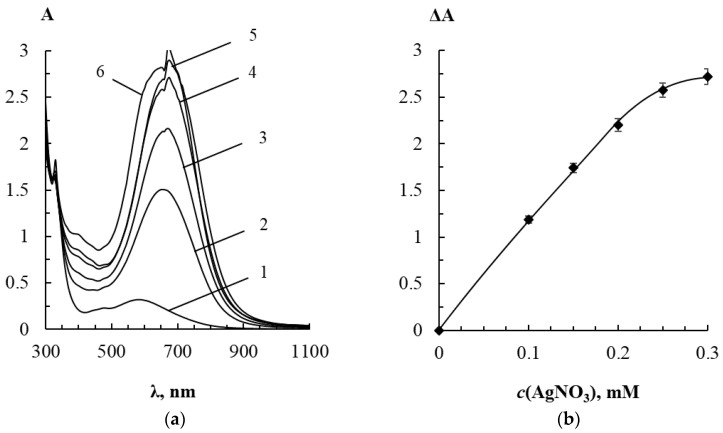
(**a**) Absorption spectra of silver triangular nanoplates before (1) and after (2–6) the interaction with quercetin in the presence of silver(I) nitrate at its different concentrations. *c*(AgNO_3_), mmol L^−1^ = 0 (1), 0.10 (2), 0.15 (3), 0.20 (4), 0.25 (5), 0.30 (6); *c*(AgTNPs) = 0.04 mmol Ag L^−1^, *c*(quercetin) = 120 μmol L^−1^, *c*(Na_2_S_2_O_5_) = 80 μmol L^−1^, pH 6.5, *t* = 20 min. (**b**) Dependence of the analytical signal (ΔA) on silver(I) nitrate concentration. *c*(AgTNPs) = 0.04 mmol Ag L^−1^, *c*(quercetin) = 120 μmol L^−1^, *c*(Na_2_S_2_O_5_) = 80 μmol L^−1^, pH 6.5, *t* = 20 min.

The absorption spectra of AgTNPs after the interaction with quercetin in the presence of different concentrations of silver(I) nitrate and the dependence of AgTNP absorbance change on silver(I) nitrate concentration are presented in Figure 8. The maximum analytical response was observed when silver(I) nitrate concentration in the reaction mixture was equal to 0.30 mmol L^−1^. Nevertheless, when comparing the absorption spectra of the different solutions, it became clear that the AgTNP LSPR band maximum corresponding to both 0.25 and 0.30 mmol L^−1^ silver(I) nitrate was fuzzy due to the presence of information noise, probably related to the UV/Vis spectrometer limitations. By contrast, the AgTNP LSPR band corresponding to 0.20 mmol L^−1^ silver(I) nitrate had a clearly shaped maximum. Moreover, a sufficiently large value of the analytical signal was also achieved in the latter case. Therefore, for further experiments, a silver(I) nitrate concentration of 0.20 mmol L^−1^ was chosen.

### 3.2. Spectrophotometric Determination of Bioflavonoids

#### 3.2.1. Analytical Performance

In the present article, we evaluated some analytical features of the developed approach to bioflavonoid determination (Table 2). Limits of detection (LODs) were evaluated using 3σ criteria, and limits of quantitation (LOQs) were assessed using 10σ criteria. We found that LODs of bioflavonoids in the selected optimal conditions increased in the series morin < rutin = quercetin < taxifolin and were equal to 0.9, 1.2, 1.2, and 2.0 μmol L^−1^, respectively. The detection range was found to be up to 120 μmol L^−1^. In all cases, the relative standard deviations (RSDs) calculated for the middle of the detection range were less than 0.04. These facts allow us to assert that the proposed approach is characterized by reasonably good sensitivity and reproducibility.

#### 3.2.2. Analytical Performance

In order to assess the selectivity of the proposed approach, solutions with different ratios of the selected bioflavonoids (namely, quercetin, morin, rutin, and taxifolin) and the extraneous compounds were prepared. The determination of analytes was carried out according to the procedure described in Section 2.3. It was found that the detection of 50 μmol L^−1^ bioflavonoid was not affected by at least 1000-fold quantities of Na^+^, K^+^, Mg^2+^, Ca^2+^, Ba^2+^, Al^3+^, Cr^3+^, Cu^2+^, Co^2+^, CH_3_COO^−^, and NO_3_^−^, and 100-fold quantities of Cl^−^, Br^−^, and SO_4_^2−^. It is interesting to note that 10-fold quantities of some reductants (such as l-ascorbic acid and glucose) also did not interfere with the bioflavonoid quantitation. These facts prove the very good selectivity of the proposed approach.

#### 3.2.3. Sample Analysis

In order to assess applicability of the developed method for the analysis of real samples, the spectrophotometric determination of bioflavonoids using AgTNPs was carried out after appropriate dilution of the stock solutions of samples. The following samples were used: Ascorutin pharmaceutical drug (Pharmstandard JSC, Dolgoprudny, Russia), ethanol tinctures of hawthorn (Flora Kavkaza JSC, Pregradnaya, Russia) and calendula (MosPharma JSC, Moscow, Russia), and onion peel. To confirm bioflavonoid determination accuracy, the studied samples were alternatively analyzed by reversed-phase high-performance liquid chromatography with amperometric detection (E = 1.2 V). The results of the proposed method represented in Table 3 are in good agreement with the results obtained using the alternative method. This indicates good accuracy of the determination.

## 4. Discussion

Analytical features of other reported methods for bioflavonoid quantitation are presented in Table 4. It is easy to conclude that the proposed approach has quite good sensitivity as it makes it possible to detect four bioflavonoids (namely, morin, rutin, quercetin, and taxifolin) with LODs lower than or at least comparable to the vast majority of the claimed spectroscopic methods. The selectivity of the proposed approach is also quite high, since a large number of components do not interfere with the determination of bioflavonoids.

In some articles to which we refer, preconcentration of analytes was used, while the proposed approach allows the determination of bioflavonoids without preconcentration or complex sample preparation. It should be stressed that we failed to find scientific articles in which the interaction between nanoparticles and bioflavonoids was studied in detail, and in which the effect of the bioflavonoid’s structure on the analytical signal was assessed. Moreover, the published papers did not consider silver triangular nanoplates as the colorimetric reagents, although their potential in optical spectroscopy methods is clearly not fully disclosed. In our opinion, the features listed above distinguish the present study favorably from existing analogues.

## 5. Conclusions

It was shown that label-free silver triangular nanoplates are a promising probe for the spectrophotometric determination of different bioflavonoids. It was found that AgTNPs undergo changes in aggregation and surface state in the presence of bioflavonoids and silver(I) nitrate. The proposed mechanism of interaction includes reducing silver(I) ions to metallic silver under the action of bioflavonoids and its deposition on the surface of AgTNPs. It was shown that the process is accompanied by aggregation of nanoparticles and leads to relatively large aggregates of irregular morphology. A low limit of 0.9–2.0 μmol L^−1^ for bioflavonoid detection could be achieved. Both the high sensitivity and the good selectivity allow for the proposed approach to be successfully applied to analyze real objects.

## Figures and Tables

**Table 1:** Bioflavonoids studied in this work.

Chemical Name	MW, g mol^−1^	Structure	lgP ^a^	E0 ^b^, V
Chrysin	254.241	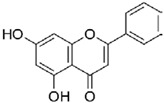	2.88	1.0 (pH 7)
Morin	302.238	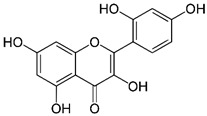	1.61	0.25 (pH 7)
Naringenin	272.256	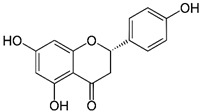	3.19	0.89 (pH 3.6)
Naringin	580.540	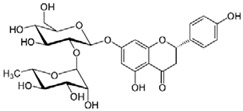	2.73	0.39 (pH 5)
Quercetin	302.236	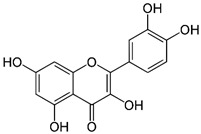	2.07	0.23 (pH 7.4)
Rutin	610.521	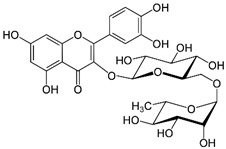	1.95	0.26 (pH 7.4)
Taxifolin	304.254	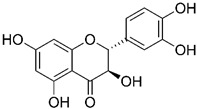	1.82	0.22 (pH 7.4)

^a^ The hydrophobicity parameters (lgP) values were calculated using the ACD Labs 6.00 software (Toronto, ON, Canada). ^b^ The E^0^ values were taken from the literature data [36,37].

**Table 2:** Analytical features of the determination of bioflavonoids.

Analyte	Calibration Curve Equation (c, μM)	LOD, μM	Determination Range, µM	RSD ^a^, %	RSD ^b^, %
Morin	ΔA = 0.0222·c (*r*^2^ = 0.997)	0.9	3–120	3	10
Rutin	ΔA = 0.0173·c (*r*^2^ = 0.999)	1.2	4–120	3	10
Quercetin	ΔA = 0.0174·c (*r*^2^ = 0.994)	1.2	4–120	3	10
Taxifolin	ΔA = 0.0099·c (*r*^2^ = 0.995)	2.0	6–120	4	11

^a^ Calculated for the middle of the determination range. ^b^ Calculated for the LOQ value.

**Table 3:** Determination of bioflavonoids in real samples (*n* = 3, *p* = 0.95).

Analyte	Sample	Present Method		Alternative Method ^a^	
		Found	RSD, %	Found	RSD, %
Quercetin	Onion peel	(38 ± 5) mg g^−1^	5.3	(44 ± 3) mg g^−1^	2.7
	Hawthorn tincture	(6.0 ± 0.9) mg L^−1^	6.0	(6.3 ± 0.5) mg L^−1^	3.2
	Calendula tincture	(17 ± 2) mg L^−1^	4.7	(18 ± 1) mg L^−1^	2.2
Rutin	Ascorutin drug	(145 ± 15) mg g^−1^	4.2	(151 ± 9) mg g^−1^	2.4

^a^ Reversed-phase high-performance liquid chromatography with amperometric detection.

**Table 4:** Comparison of the AgTNP-based method with other spectroscopic methods for the determination of bioflavonoids.

Analyte	Method	LOD, μM	Determination Range, μM	Reference
Morin	Diffuse reflectance spectroscopy	17	53–330	[2]
	Fluorimetry	0.3	14.5–32.5	[38]
	Present method	0.9	3–120	This study
Quercetin	Diffuse reflectance spectroscopy	8	25–330	[2]
	Spectrophotometry	0.23	-	[39]
	Present method	1.2	4–120	This study
Rutin	Fluorimetry	0.2	0.5–15	[40]
	Present method	1.2	4–120	This study
Taxifolin	Diffuse reflectance spectroscopy	46	142–330	[2]
	Present method	2.0	6–120	This study

## Data Availability

Not applicable.
